# Association of a high-protein and low-glycemic-index diet during pregnancy with offspring growth and obesity until the age of 18 years – a target trial emulation

**DOI:** 10.1038/s41430-025-01666-2

**Published:** 2025-09-25

**Authors:** Christina Sonne Mogensen, Christian Mølgaard, Faidon Magkos, Nina Rica Wium Geiker, Anne Ahrendt Bjerregaard, Charlotta Granström, Thorhallur Ingi Halldorsson, Sjurdur Frodi Olsen

**Affiliations:** 1 Department of Nutrition, Exercise and Sports, Faculty of Science, University of Copenhagen (UCPH), Copenhagen, Denmark; 2 Centre for Childhood Health, Copenhagen, Denmark; 3 Department of Epidemiology Research, Statens Serum Institut, Copenhagen, Denmark; 4 Section of Epidemiology, Frederiksberg Hospital Center for Klinisk Forskning og Forebyggelse, Frederiksberg, Denmark; 5 Faculty of Food Science and Nutrition, School of Health Sciences, University of Iceland, Reykjavik, Iceland; 6 Department of Public Health, University of Copenhagen, 1353 Copenhagen, Denmark; 7 Harvard TH Chan School of Public Health, Boston, MA USA; 8 University of the Faroe Islands, J. C. Svabos gøta 14, Torshavn, Faroe Islands

**Keywords:** Risk factors, Nutrition, Epidemiology

## Abstract

**Background:**

Maternal pre-pregnancy BMI and excessive gestational weight gain (GWG) are associated with offspring obesity. Moreover, maternal dietary patterns, particularly protein intake and glycemic index, influence GWG and fetal development. This study aimed to investigate the association of a high-protein, low-glycemic-index (HPLGI) diet during pregnancy with offspring growth and obesity.

**Methods:**

Using observational data from the Danish National Birth Cohort, a target trial emulation was conducted to investigate the association of an HPLGI diet during pregnancy on offspring growth up to 18 years of age. A cohort of 17,551 women who met the inclusion criteria was categorized into exposure groups based on their protein intake and glycemic index to emulate the groups in the APPROACH trial.

**Results:**

Maternal characteristics varied between the exposure groups, with the HPLGI women exhibiting 1.67 kg higher pre-pregnancy weight and 0.49 kg/m^2^ BMI, with no differences in GWG. Offspring outcomes were assessed at various ages, and a linear mixed model was used, including potential confounders. Offspring born to women following an HPLGI diet during pregnancy had 2.59 kg higher body weight and 0.72 kg/m^2^ higher BMI at 18 years compared to those from the moderate-protein, moderate-glycemic-index (MPMGI) group.

**Conclusion:**

An HPLGI diet during pregnancy was associated with higher offspring body weight and BMI at 18 years of age compared to an MPMGI diet. These findings suggest that maternal dietary composition during pregnancy may have long-term implications for offspring growth and obesity risk, underscoring the importance of evaluating dietary recommendations during pregnancy.

## Introduction

Individuals with obesity are at higher risk for developing many non-communicable diseases, such as type 2 diabetes, heart disease, and cancer [1]. In Denmark, about 50% of adults and 20% of primary school-aged children are classified as overweight or obese [2, 3]. Globally, the prevalence of childhood obesity has tripled since the 1990s, and children who are overweight are significantly more prone to becoming adults with obesity [4]. Preventing childhood obesity may thus have widespread implications for non-communicable diseases in the whole population.

Pre-pregnancy overweight and excessive gestational weight gain (GWG) increase the risk of the offspring being born large-for-gestational-age and developing overweight later in life [5]. Despite the recommendation for GWG below 9 kg for women with pre-pregnancy obesity, almost 60% of them exceed this threshold [6]. The characteristics of the maternal diet, including low dietary protein intake and low protein-to-carbohydrate ratio, appear to be associated with increased GWG, fetal adiposity, and fetal abdominal fat deposition [7–9]. Research in non-pregnant individuals exploring diets with varying protein-to-carbohydrate ratios and glycemic index (GI) has highlighted the efficacy of a high-protein diet with a low GI in preventing weight regain after weight loss [10]. In pregnant women, high GI diets are associated with an increased risk of excessive GWG, postnatal weight retention, and delivery of large-for-gestational-age infants, who are likely to become adults with elevated BMI [11–13]. Throughout pregnancy, the maternal diet serves as the sole source for the developing fetus. Nevertheless, conflicting results exist regarding the association between maternal protein intake and offspring birth weight, with some studies reporting a positive association [14] and others not [15, 16]. Additionally, studies on maternal protein intake during pregnancy and offspring later weight are also inconclusive [17, 18].

An intervention study—the APPROACH trial—targeting higher protein and lower GI intake among well-nourished overweight and obese pregnant women found significant effects on lower GWG (1.7 kg) [19] and a positive association between GWG and offspring BMI z-score at birth [20]. However, no effect of the intervention diet was found on offspring BMI until the age of 5 years but showed some indication of worse metabolic profile in the offspring compared to controls [21]. Here, we utilize data from the large Danish National Birth Cohort (DNBC) to conduct an emulation of the APPROACH trial. Our aim is to replicate the dietary regimens employed in the APPROACH trial for the purpose of investigating the long-term association of an HPLGI diet during pregnancy on offspring growth up until the age of 18 years within an observational setting of DNBC.

## Methods

We first specified the protocol and developed a data analysis strategy for our target trial emulation. Key components are described in Table 1.

**Table 1:** Target Trial Emulation of the APPROACH dietary intervention study.

PROTOCOL	APPROACH RCT	TARGET TRIAL EMULATION
**ELIGIBILITY CRITERIA**	Pre-pregnancy BMI 28-45 Older 18–45 years Singleton pregnancy No abuse of alcohol or drugs ( >14 units of alcohol per week) No critical or chronic diseases	Pre-pregnancy BMI 25–45 Older than 18 years Singleton pregnancy No abuse of alcohol or drugs ( >14 units of alcohol per week) Plausible total energy intakes ( >2500 kJ/day and <25,000 kJ/day)
**TREATMENT STRATEGY**	High-protein (25–28 E%), and low-GI (GI <55) ad libitum diet. Active Comparator: Moderate-protein (protein 18 E%) moderate-GI (no information on restricting glycemic load but guided following the principles of the New Nordic Diet (GI ~60)) ad libitum diet.	High-protein ( ≥18 E%) and low-GI (GI ≤55) ad libitum diet. Active Comparator: Moderate-protein (protein <18 E%) moderate-GI (GI >55-100) ad libitum diet.
**RANDOMIZATION**	Participants were randomly assigned in gestational week 15 in clusters of 4–8 women to either the HPLGI diet or the MPMGI diet.	We assumed randomization conditional on the eligibility criteria and the exposure diet, and adjusted for covariates: maternal pre-pregnancy BMI, SES, GWG, parity, offspring sex, and offspring gestational age.
**OUTCOME**	Primary outcome: Gestational weight gain Secondary outcome: Growth and development of the fetus and child from birth to 9 years of age.	Primary outcome: Growth and development of the fetus and child from birth to 18 years of age. Secondary outcome: Gestational weight gain
**FOLLOW-UP**	Starts after the randomization in 2013 and ends at the last follow-up visit at 7-9 years of age, expected in 2025.	Starts after the dietary questionnaires in 1996 and 2003 and ends with the follow-up of the offspring at 18 years.
**CAUSAL CONTRAST**	Intention to treat effect; per-protocol effect.	Observational analog of per-protocol effect.
**ANALYSIS PLAN**	Available case analyses of the follow-up of the offspring. The linear mixed model methodology with offspring age as a fixed effect and participant-specific ID as a random effect was used to assess the difference between the HPLGI diet and the MPMGI diet on offspring outcomes.	Same as the APPROACH trial + pre-assignment data, including the eligibility criteria and thresholds for the exposure group and post-assignment confounders such as maternal pre-pregnancy BMI, SES, GWG, parity, offspring sex, and offspring gestational age.

### Study design and setting

The DNBC is a large prospective cohort study, which enrolled almost 95,000 mother-offspring dyads between 1996 and 2003. During pregnancy, the women completed a food frequency questionnaire (FFQ) in gestational week (GW) 25 [22]. The women gave written informed consent on behalf of themselves and their offspring. The DNBC was conducted according to the guidelines in the Declaration of Helsinki, and the National Committee on Health Research Ethics in Denmark (H-4-2011-045) approved all procedures.

### Participants

**Fig. 1:**
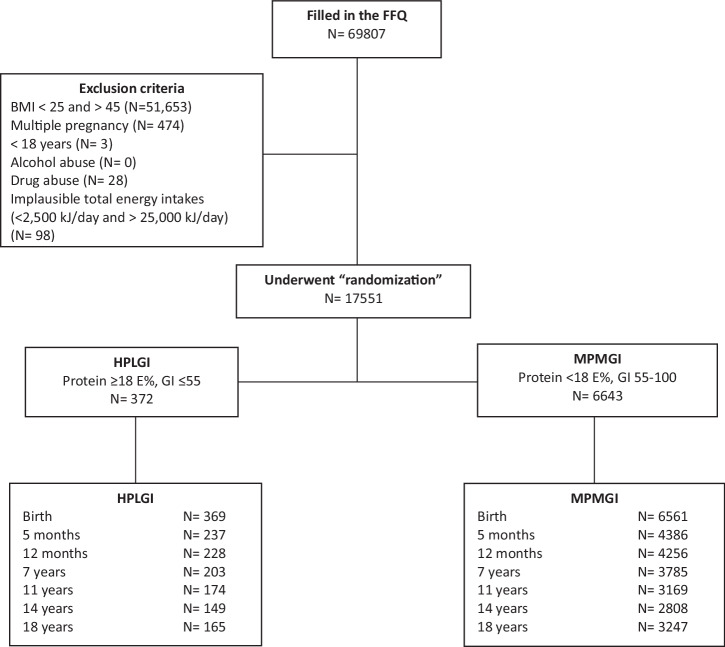
Flow chart. FFQ food frequency questionnaire. HPLGI high-protein low-glycemic-index. MPMGI moderate-protein moderate-glycemic-index.

Among participating women in the DNBC cohort, 69,807 had information on nutrient intake from the FFQ administered in GW 25 (Fig. 1). From these, we selected a sub-cohort based on the APPROACH randomized controlled trial (RCT) inclusion and exclusion criteria: BMI 28–45 kg/m^2^; singleton birth; older than 18 years; and no alcohol or drug abuse. Additionally, we excluded implausible total energy intakes ( <2500 kJ/day and >25,000 kJ/day) ( ~1.0%). All the inclusion criteria were met by 17,551 women (25.1%).

### Modifications of the target trial protocol, to adapt the analyses to the observational setting within DNBC

We applied the design principles of the target trial methodology [23] to replicate the exposure groups of the APPROACH RCT and prolong the follow-up period for offspring growth until the age of 18 years. Nevertheless, we made necessary modifications to the trial protocol, such as a lower protein target and maternal BMI compared to the original trial protocol, to enhance the statistical power of our analysis, while still addressing the same primary objective as the APPROACH RCT (Table 1). The APPROACH RCT excluded women with a chronic disease, such as cancer, diabetes, or gestational diabetes mellitus. However, given the low prevalence rates of chronic diseases in the DNBC cohort, e.g., gestational diabetes mellitus [24], individuals with these conditions were not excluded from the analysis, as their inclusion would not significantly influence our results. Furthermore, we attempted to emulate randomization through the eligibility criteria and by adjusting for confounding variables.

### Exposure assessment

A 360-item FFQ was used to collect information on maternal diet in GW 25. All frequencies were computed into times per day. To estimate the intake of each food item in grams, standard portion sizes were multiplied by the daily frequencies. The FFQ had three main components: food frequency, dietary supplements, and other information. The questionnaire was validated against 7-day food diaries and urine nitrogen excretion (*n* = 88) [25] and developed from the questionnaire used by the Danish Cancer Registry [26]. Specific GI values for each food-item in the FFQ were extracted from published data [27, 28] and were calculated by multiplying available carbohydrates (in grams) for the food item with the GI of the specific food item, summing up, and dividing by the total intake of available carbohydrates. White bread was used as a reference.

### Exposure groups

Before the authors had access to outcomes, two exposure groups were defined based on maternal protein intake and GI. According to the hypothesis of the APPROACH RCT, we defined a high-protein low-glycemic index (HPLGI) group as protein ≥18 percent of total energy intake (E%) and GI ≤ 55, and a moderate-protein moderate glycemic index (MPMGI) group as protein <18 E% and GI >55-100. The chosen GI cut-off was determined by the specified threshold in the APPROACH RCT, and the International Standard Organization categorization for low GI [27]. For the MPMGI group, the GI was set to range from 55 to 100 to facilitate differences in GI between the two groups. The protein intake cut-off was selected considering the Nordic nutrition recommendations (dietary protein 10–20 E%) [29], and the high-protein threshold was then set at ≥18 E%. The final exposure groups consisted of 372 mother-offspring dyads in the HPLGI group and 6643 mother-offspring dyads in the MPMGI group (Fig. 1).

### Outcome variables

Breastfeeding and GWG were obtained from self-reported questionnaires, 6 months postpartum. GWG was defined based on the questions: “*How many kilograms did you gain or lose throughout your pregnancy?*” and “How many kilograms did you lose throughout your pregnancy?”. Breastfeeding duration was defined as the duration of any breastfeeding, not distinguishing between exclusive and supplemented breastfeeding, in categories of <1, 1 to <4, 4 to <6, or ≥6 months. Smoking during pregnancy was obtained on telephone interviews in GW 12 and GW 30 (Daily <15 cigarettes, Daily ≥15 cigarettes, Occasionally, or Non-smoker). Offspring weight and length were obtained from The National Patient Registry at birth, 5 months of age, and 12 months of age. Moreover, offspring weight and height were obtained at ages 7 years, 11 years, 14 years, and 18 years by questionnaires. Age- and sex-specific BMI z-scores were calculated by using the WHO Child Growth Standards for offspring aged <5 years and the WHO Growth Reference for those ≥5 years [30, 31]. We further classified the offspring as overweight/obese by using the corresponding age- and sex-specific WHO cutoffs [i.e., 1-1.99 SD as overweight and ≥2 SD as obese] [32].

### Covariate assessment

Based on telephone interviews in GW 12 and GW 30 information on the following set of covariates included in our analyses were recorded: Parity (nulliparous or not), socioeconomic status (based on the highest level of professional occupation within the couple and categorized as “high” including high- or medium-level professionals, or skilled workers, or “low” including unskilled workers and others,), pre-pregnancy BMI (calculated as self-reported pre-pregnancy weight in kilograms divided by height in meters squared and categorized into overweight: BMI 25–29.9 kg/m^2^ and obese: BMI ≥ 30 kg/m^2^). Information on the offspring’s gestational age at delivery (days) was extracted from medical records.

### Statistics

To ensure that the sample size was adequate for comparing two means with a two-sided test, a power calculation was conducted. With a study population of 369 offspring at birth in the HPLGI group and 6561 offspring in the MPMGI group, and an estimated SD for BMI z-score of 1.16 [33], the difference in BMI z-score between the groups needed to be at least 0.174 SD to be detected with an 80% probability at a *p*-value of 0.05.

Maternal characteristics are presented as mean (SD) for continuous variables and frequencies for categorical variables. Differences between the HPLGI group and the MPMGI group were analyzed using ANOVA. To assess the differences in offspring outcomes between the HPLGI and MPMGI groups during the first 18 years of life, we conducted an observational analog of a per-protocol analysis, using a linear mixed model with offspring age (birth, 5 months, 12 months, 7 years, 11 years, 14 years, and 18 years) as a fixed effect and participant-specific ID as a random effect. We conducted three models: an unadjusted model (Model 1), a model adjusted for maternal pre-pregnancy BMI, SES, parity, and offspring sex (Model 2), and a fully adjusted model that additionally accounted for GWG and gestational age (Model 3). The residuals of all the models described above were investigated to assess normality. The Sidak correction was used to adjust the significance level for multiple comparisons over time within the group and presented as p-adjusted.

## Results

### Study participants

Among the 69,807 pregnant women, 17,551 (25.1%) met the inclusion/exclusion criteria. Among these, 372 women were assigned to the HPLGI diet group, and 6643 women were assigned to the MPMGI diet group. Follow-up assessments of offspring growth were completed from birth to 18 years of age, with a follow-up rate from 99% at birth to ~46% at 18 years of age (Fig. 1). Women who dropped out between their offspring’s birth and age of 18 years were, on average, 0.33 years younger (30.3 years ± 4.23) compared to those whose children completed the 18-year questionnaire (30.6 years ± 4.08) (*P* <0.001). The mothers were similar in terms of weight, height, pre-pregnancy BMI, GWG, energy intake, protein intake (both percentage and grams per day), and glycemic index. Additionally, their offspring had comparable birthweights and BMI z-scores (Table S1).

Women in the HPLGI group had higher pre-pregnancy weight (1.67 kg, 95% CI: 0.46; 2.88) and higher pre-pregnancy BMI (0.49 kg/m^2^, 95% CI: 0.11; 0.86) compared to those in the MPMGI group. Additionally, women who consumed an HPLGI diet had on average 2774 (95% CI: -2998; -2549) kJ/day lower energy intake compared to those in the MPMGI group. GWG was equal among the two groups (*P* = 0.134) (Table 2).

**Table 2:** Maternal characteristics.

	*N*	HPLGI (*N* = 372)	*N*	MPMGI (*N* = 6643)	(HPLGI-MPMGI Estimate)	*P*-value
**Maternal age at delivery (years)**	372	30.4 (4.06)	6643	30.5 (4.17)	−0.06 (−0.50; 0.37)	0.776
**Gestational age (days)**	372	280.5 (12.4)	6643	280.9 (12.5)	−0.42 (−1.73; 0.88)	0.526
**Pre-pregnancy weight (kg)**	372	83.3 (11.6)	6643	81.6 (11.6)	1.67 (0.46; 2.88)	**0.007**
**Pre-pregnancy height (cm)**	372	168.8 (6.33)	6643	168.5 (6.05)	0.36 (−0.27; 0.99)	0.266
**Pre-pregnancy BMI (kg/m**^**2**^**)**	372	29.2 (3.73)	6643	28.7 (3.56)	0.49 (0.11; 0.86)	**0.010**
**BMI categories**						**0.040**
Overweight (%)	250	67.2	4801	72.3		
Obese (%)	122	32.8	1842	27.7		
**GWG (kg)**	291	12.88 (7.27)	5425	13.52 (7.03)	−0.64 (−1.47; 0.20)	0.134
**Parity (%)**						0.144
0	182	48.9	2916	43.9		
1	130	34.9	2618	39.4		
2+	60	16.1	1108	16.7		
**Breastfeeding (months)**						0.981
≤1	67	46.2	1378	46.3		
1–4	32	22.1	670	22.5		
4–6	35	24.1	731	24.5		
6+	11	7.6	200	6.7		
**Smoking (%)**						0.574
Daily <15 cigarettes	34	9.1	664	10.0		
Daily 15+ cigarettes	8	2.2	135	2.0		
Occasionally	49	13.2	726	10.9		
None smoker	281	75.5	5118	77.0		
**Socioeconomic status (%)**						**0.016**
High	315	84.9	5276	79.6		
Low	56	15.1	1349	20.4		
**Energy intake (kJ)**	372	7139 (1643)	6643	9912 (2174)	−2774 (−2998;−2549)	**<0.001**
**Protein intake (%)**	372	19.3 (1.22)	6643	14.9 (1.75)	4.35 (4.17; 4.53)	**<0.001**
**Fat intake (%)**	372	30.5 (5.49)	6643	30.6 (5.74)	−0.18 (−0.78; 0.41)	0.547
**Carbohydrate (%)**	372	49.8 (5.87)	6643	54.0 (5.60)	−4.24 (−4.82;−3.65)	**<0.001**
Fiber (g/day)	372	16.5 (6.51)	6643	26.1 (7.72)	−9.52 (−10.3;−8.72)	**<0.001**
Added sugar (g/day)	372	25.4 (11.7)	6643	45.8 (24,8)	−20.38 (−22.9;−17.8)	**<0.001**
**Glycemic index (pr. day)**	372	42.8 (10.95)	6643	76.0 (13.02)	−33.19 (−34.54;−31.84)	**<0.001**

*GWG* Gestational weight gain, *HPLGI* High-protein low-glycemic-index, *MPMGI* moderate-protein moderate-glycemic-index, *BMI* Body mass index. Data are presented as mean (standard deviation (SD)) for continuous variables or as numbers (%) for categorical variables. Bold indicates a significant difference from the MPMGI group.

### Offspring outcomes

At 18 years of age, offspring born to women who consumed an HPLGI diet had, on average, 2.59 (95% CI: 1.31;3.87) kg higher body weight and 0.72 (95% CI: 0.30;1.14) kg/m^2^ higher BMI compared with offspring born to women in the MPMGI group (Table 3**, Model 3**). No differences between the HPLGI group and the MPMGI group on offspring outcomes from birth to 14 years of age were found after adjusting the *p*-value for multiple comparisons.

**Table 3:** Offspring characteristics.

	Model 1					Model 2					Model 3				
	HPLGI	MPMGI	(HPLGI-MPMGI Estimate)	*P*-value	*P-adjust	HPLGI	MPMGI	(HPLGI-MPMGI Estimate)	*P*-value	*P-adjust	HPLGI	MPMGI	(HPLGI-MPMGI Estimate)	*P*-value	*P-adjust
**Body weight, kg**															
Birth	3.7 (0.38)	3.7 (0.09)	−0.04 (−0.80;0.73)	0.921	1.000	3.6 (0.40)	3.7 (0.10)	−0.16 (−0.92;0.60)	0.677	1.000	3.5 (0.43)	3.7 (0.11)	−0.18 (−1.04;0.67)	0.673	1.000
5 months	7.9 (0.46)	7.9 (0.11)	0.01 (−0.93;0.94)	0.991	1.000	7.8 (0.46)	8.0 (0.12)	−0.13 (−1.05;0.80)	0.788	1.000	7.9 (0.52)	7.9 (0.13)	−0.08 (−1.11;0.96)	0.887	1.000
12 months	10.5 (0.47)	10.4 (0.11)	0.09 (−0.86;1.04)	0.851	1.000	10.4 (0.47)	10.4 (0.12)	−0.07 (−1.01;0.87)	0.884	1.000	10.4 (0.53)	10.4 (0.13)	−0.01 (−1.07;1.05)	0.986	1.000
7 years	25.9 (0.50)	25.9 (0.12)	−0.03 (−1.04;0.97)	0.950	1.000	25.9 (0.50)	26.0 (0.12)	−0.15 (−1.15;0.85)	0.766	1.000	26.0 (0.56)	25.9 (0.14)	0.03 (−1.09;1.14)	0.964	1.000
11 years	41.6 (0.54)	42.8 (0.13)	−1.21 (−2.29;−0.13)	**0.028**	0.181	41.6 (0.54)	42.9 (0.13)	−1.31 (−2.38;−0.24)	**0.017**	0.113	41.5 (0.60)	42.4 (0.15)	−0.95 (−2.16;0.26)	0.122	0.599
14 years	58.8 (0.58)	58.5 (0.13)	0.27 (−0.90;1.43)	0.653	0.999	58.8 (0.58)	58.6 (0.14)	0.20 (−0.95;1.36)	0.731	1.000	58.9 (0.64)	58.5 (0.15)	0.40 (−0.90; 1.65)	0.562	0.997
18 years	73.8 (0.55)	73.4 (0.13)	0.48 (−0.63;1.58)	0.397	0.971	73.9 (0.55)	73.5 (0.13)	0.41 (−0.69;1.51)	0.468	0.988	76.1 (0.64)	73.5 (0.14)	2.59 (1.31;3.87)	**<.0001**	**<0.001**
**Body length/height, cm**															
Birth	52.5 (0.30)	52.6 (0.07)	−0.07 (−0.67;0.52)	0.812	1.000	52.3 (0.29)	52.5 (0.08)	−0.18 (−0.76;0.40)	0.541	0.906	52.4 (0.33)	52.5 (0.08)	−0.14 (−0.79;0.50)	0.663	1.000
5 months	68.5 (0.35)	68.3 (0.08)	0.18 (−0.53;0.89)	0.624	0.999	68.3 (0.35)	68.2 (0.09)	0.08 (−0.61;0.78)	0.818	1.000	68.4 (0.39)	68.2 (0.10)	0.16 (−0.61;0.93)	0.685	1.000
12 months	77.5 (0.36)	77.6 (0.08)	−0.12 (−0.85;0.60)	0.736	1.000	77.3 (0.35)	77.5 (0.09)	−0.23 (−0.93;0.48)	0.533	0.995	77.5 (0.39)	77.6 (0.10)	−0.09 (−0.88;0.69)	0.819	1.000
7 years	126.5 (0.38)	126.1 (0.09)	0.44 (−0.32;1.19)	0.258	0.877	126.4 (0.37)	126.0 (0.09)	0.36 (−0.38;1.10)	0.341	0.946	126.5 (0.41)	125.9 (0.10)	0.57 (−0.24;1.39)	0.168	0.724
11 years	151.3 (040)	151.5 (0.09)	−0.27 (−1.07;0.53)	0.504	0.993	151.1 (0.39)	151.5 (0.10)	−0.39 (−1.12;0.44)	0.396	0.971	151.1 (0.44)	151.0 (0.11)	0.03 (−0.84;0.91)	0.944	1.000
14 years	169.5 (0.43)	168.8 (0.10)	0.64 (−0.22;1.51)	0.144	0.663	169.4 (0.42)	168.8 (0.10)	0.62 (−0.22;1.47)	0.149	0.677	169.5 (0.46)	168.7 (0.11)	0.77 (−0.15;1.69)	0.101	0.526
18 years	174.9 (0.41)	174.9 (0.09)	−0.04 (−0.86;0.79)	0.928	1.000	174.9 (0.41)	174.9 (0.10)	−0.03 (−0.84;0.78)	0.949	1.000	175.4 (0.47)	175.0 (0.11)	0.33 (−0.60;1.26)	0.484	0.990
**BMI**															
Birth	13.16 (0.13)	13.28 (0.03)	−0.12 (−0.37;0.14)	0.364	0.958	13.16 (0.13)	13.29 (0.03)	−0.13 (−0.38;0.13)	0.320	0.933	13.12 (0.14)	13.30 (0.04)	−0.19 (−0.47;0.10)	0.204	0.798
5 months	13.15 (0.15)	13.32 (0.04)	−0.16 (−0.47;0.15)	0.304	0.921	13.15 (0.15)	13.32 (0.04)	−0.17 (−0.48;0.14)	0.275	0.895	13.11 (0.17)	13.33 (0.04)	−0.22 (−0.56;0.13)	0.224	0.830
12 months	13.17 (0.16)	13.31 (0.04)	−0.14 (−0.45;0.18)	0.399	0.972	13.16 (0.16)	13.31 (0.04)	−0.16 (−0.47;0.16)	0.332	0.941	13.11 (0.18)	13.32 (0.04)	−0.21 (−0.56;0.14)	0.238	0.851
7 years	13.13 (0.16)	13.34 (0.04)	−0.21 (−0.54;0.12)	0.203	0.796	13.13 (0.16)	13.36 (0.04)	−0.23 (−0.56;0.10)	0.172	0.733	13.10 (0.18)	13.36 (0.04)	−0.27 (−0.63;0.10)	0.153	0.688
11 years	18.08 (0.18)	18.51 (0.04)	−0.43 (−0.78;−0.08)	**0.017**	0.115	18.08 (0.18)	18.52 (0.04)	−0.44 (−0.79;−0.09)	**0.015**	0.097	18.11 (0.20)	18.44 (0.05)	−0.33 (−0.73;0.07)	0.104	0.535
14 years	20.43 (0.19)	20.47 (0.04)	−0.04 (−0.42;0.34)	0.846	1.000	20.43 (0.19)	20.48 (0.05)	−0.46 (−0.42;0.33)	0.812	1.000	20.47 (0.21)	20.47 (0.05)	−0.01 (−0.42;0.41)	0.982	1.000
18 years	24.06 (0.18)	23.93 (0.04)	0.13 (−0.23;0.49)	0.487	0.991	24.04 (0.18)	23.94 (0.04)	0.11 (−0.25;0.47)	0.559	0.997	24.64 (0.21)	23.92 (0.05)	0.72 (0.30;1.14)	**0.001**	**0.005**
**BMI z−score (0−228 months)**															
Birth	−0.24 (0.06)	−0.14 (0.01)	−0.10 (−0.02;0.22)	0.095	0.501	−0.24 (0.06)	−0.14 (0.02)	−0.11 (−0.23;0.01)	0.075	0.419	−0.28 (0.07)	−0.12 (0.02)	−0.17 (−0.30;−0.03)	**0.015**	0.102
5 months	−0.31 (0.07)	−0.23 (0.02)	−0.08 (−0.06;0.23)	0.263	0.882	−0.31 (0.08)	−0.22 (0.02)	−0.09 (−0.23;0.06)	0.235	0.847	−0.28 (0.08)	−0.24 (0.02)	0.04 (−0.20;0.12)	0.606	0.999
12 months	0.51 (0.07)	0.39 (0.02)	0.13 (−0.28;0.02)	0.084	0.460	0.50 (0.07)	0.39 (0.02)	0.11 (−0.03;0.26)	0.131	0.625	0.52 (0.08)	0.38 (0.02)	0.14 (−0.02;0.30)	0.093	0.496
7 years	0.29 (0.08)	0.32 (0.02)	−0.03 (−0.12;0.19)	0.686	1.000	0.30 (0.08)	0.33 (0.02)	−0.03 (−0.18;0.12)	0.685	1.000	0.32 (0.09)	0.32 (0.02)	−0.00 (−0.17;0.17)	0.966	1.000
11 years	0.16 (0.08)	0.31 (0.02)	−0.15 (−0.02;0.31)	0.081	0.448	0.16 (0.08)	0.32 (0.02)	−0.15 (−0.32;0.01)	0.070	0.397	0.20 (0.09)	0.31 (0.02)	−0.11 (−0.29;0.08)	0.262	0.881
14 years	0.21 (0.09)	0.20 (0.02)	0.01 (−0.19; 0.17)	0.909	1.000	0.22 (0.09)	0.21 (0.02)	0.01 (−0.17;0.18)	0.945	1.000	0.21 (0.10)	0.21 (0.02)	0.01 (−0.19;0.19)	0.940	1.000
18 years	0.60 (0.08)	0.54 (0.02)	0.06 (−0.23; 0.11)	0.481	0.990	0.60 (0.08)	0.55 (0.02)	0.05 (−0.11;0.22)	0.532	0.995	0.75 (0.10)	0.54 (0.02)	0.20 (0.01:0.40)	**0.040**	0.248
**Weight z−score (0−120 months)**															
Birth	0.67 (0.06)	0.75 (0.01)	−0.08 (−0.19;0.03)	0.150	0.479	0.65 (0.06)	0.74 (0.02)	−0.09 (−0.20;0.03)	0.131	0.429	0.65 (0.06)	0.76 (0.02)	−0.11 (−0.23;0.01)	0.068	0.247
5 months	0.51 (0.07)	0.57 (0.02)	−0.06 (−0.19;0.08)	0.416	0.883	0.50 (0.07)	0.55 (0.02)	−0.05 (−0.19;0.08)	0.423	0.889	0.56 (0.07)	0.56 (0.02)	0.00 (−0.14;0.14)	0.979	1.000
12 months	0.81 (0.07)	0.79 (0.02)	0.02 (−0.11;0.16)	0.753	0.996	0.79 (0.07)	0.77 (0.02)	0.02 (−0.12;0.15)	0.810	0.999	0.85 (0.07)	0.77 (0.02)	0.08 (−0.07;0.22)	0.302	0.762
7 years	0.79 (0.07)	0.73 (0.02)	0.05 (−0.09;0.19)	0.465	0.918	0.77 (0.07)	0.72 (0.02)	0.05 (−0.09;0.19)	0.450	0.909	0.81 (0.07)	0.72 (0.02)	0.09 (−0.06;0.24)	0.228	0.645
**Length z−score (0−228 months)**															
Birth	1.58 (0.06)	1.63 (0.01)	−0.05 (−0.17;0.07)	0.435	0.982	1.56 (0.06)	1.61 (0.02)	−0.05 (−0.17;0.07)	0.396	0.971	1.61 (0.07)	1.64 (0.02)	−0.03 (−0.16;0.11)	0.755	1.000
5 months	1.33 (0.07)	1.33 (0.02)	0.00 (−0.14;0.15)	0.966	1.000	1.31 (0.07)	1.31 (0.02)	0.01 (−0.14;0.15)	0.938	1.000	1.36 (0.08)	1.33 (0.02)	0.03 (−0.12;0.19)	0.649	1.000
12 months	0.80 (0.07)	0.91 (0.02)	−0.11 (−0.25;0.03)	0.133	0.631	0.78 (0.07)	0.89 (0.02)	−0.11 (−0.25;0.04)	0.147	0.671	0.85 (0.08)	0.90 (0.02)	−0.05 (−0.21;0.11)	0.558	0.994
7 years	0.96 (0.07)	0.86 (0.02)	0.11 (−0.04;0.26)	0.164	0.715	0.94 (0.08)	0.84 (0.02)	0.10 (−0.05;0.25)	0.175	0.741	0.97 (0.08)	0.85 (0.02)	0.12 (−0.04;0.29)	0.127	0.653
11 years	0.83 (0.08)	0.79 (0.02)	0.04 (−0.11;0.20)	0.578	0.998	0.81 (0.08)	0.77 (0.02)	0.05 (−0.11;0.20)	0.575	0.998	0.86 (0.09)	0.77 (0.02)	0.09 (−0.09;0.26)	0.312	0.941
14 years	0.93 (0.08)	0.88 (0.02)	0.05 (−0.12;0.22)	0.552	0.996	0.92 (0.08)	0.86 (0.02)	0.05 (−0.12;0.22)	0.545	0.996	0.96 (0.09)	0.87 (0.02)	0.09 (−0.09;0.27)	0.336	0.950
18 years	0.84 (0.08)	0.90 (0.02)	−0.06 (−0.23;0.10)	0.446	0.984	0.83 (0.08)	0.89 (0.02)	−0.06 (−0.22;0.10)	0.481	0.990	0.84 (0.09)	0.90 (0.02)	−0.05 (−0.24;0.13)	0.606	0.997

Data are presented as estimated means (SE). The difference between groups is presented as the estimated means from the HPLGI-MPMGI with 95% confidence intervals (CI). HPLGI: high-protein low–glycemic index; MPMGI: moderate-protein moderate–glycemic index. (Model 1) unadjusted, (model 2) adjusted for pre-pregnancy BMI, parity, socioeconomic status, and offspring sex, (Model 3) Model 2 + GWG and gestational age. *Adjusted with the Sidak correction. Bold indicates a significant difference from the MPMGI group.

Moreover, using the WHO age- and sex-specific categories for underweight, normal weight, overweight, or obesity, we found no difference between the groups at different ages (Table 4).

**Table 4:** Obesity categories.

	HPLGI	MPMGI	*P*-value
**Birth**			0.900
Underweight, *n* (%)	19 (5.15)	354 (5.40)	
Normal weight, *n* (%)	344 (93.23)	6095 (92.89)	
Overweight, *n* (%)	6 (1.63)	100 (1.52)	
Obese, *n* (%)	0 (0.00)	12 (0.18)	
**5 months**			0.800
Underweight, *n* (%)	17 (7.17)	250 (5.70)	
Normal weight, *n* (%)	214 (90.30)	4025 (91.77)	
Overweight, *n* (%)	5 (2.11)	93 (2.12)	
Obese, *n* (%)	1 (0.42)	18 (0.41)	
**12 months**			0.700
Underweight, *n* (%)	4 (1.75)	87 (2.04)	
Normal weight, *n* (%)	208 (91.23)	3872 (90.98)	
Overweight, *n* (%)	12 (5.26)	253 (5.94)	
Obese, *n* (%)	4 (1.75)	44 (1.03)	
**7 years**			0.400
Underweight, *n* (%)	3 (1.46)	62 (1.63)	
Normal weight, *n* (%)	148 (72.2)	2796 (73.50)	
Overweight, *n* (%)	46 (22.44)	711 (18.69)	
Obese, *n* (%)	8 (3.90)	235 (6.18)	
**11 years**			0.300
Underweight, *n* (%)	7 (4.02)	84 (2.65)	
Normal weight, *n* (%)	127 (72.99)	2201 (69.45)	
Overweight, *n* (%)	29 (16.67)	686 (21.65)	
Obese, *n* (%)	11 (6.32)	198 (6.25)	
**14 years**			0.900
Underweight, *n* (%)	2 (1.34)	61 (2.17)	
Normal weight, *n* (%)	115 (77.18)	2134 (76.00)	
Overweight, *n* (%)	26 (17.45)	499 (17.77)	
Obese, *n* (%)	6 (4.03)	114 (4.06)	
**18 years**			0.500
Underweight, *n* (%)	3 (1.82)	43 (1.32)	
Normal weight, *n* (%)	108 (65.45)	2208 (68.00)	
Overweight, *n* (%)	40 (24.24)	657 (20.23)	
Obese, *n* (%)	14 (10.44)	339 (8.48)	

Data are presented as numbers (%) of participants in each obesity category; underweight, normal weight, overweight, or obese. *HPLGI* high-protein low–glycemic index, *MPMGI* moderate-protein moderate–glycemic index.

## Discussion

This population-based cohort study found that offspring born to women who consumed an HPLGI diet during pregnancy exhibited higher body weight and BMI at 18 years of age compared to those born to women who consumed an MPMGI diet during pregnancy. Moreover, offspring from the HPLGI group had a higher BMI z-score at 18 years, but this difference disappeared after adjusting the *p*-value for multiple comparisons.

In the APPROACH RCT, offspring born to women who consumed an HPLGI diet during their pregnancy exhibited unfavorable effects in their lipid blood markers at 3 and 5 years of age compared with offspring born to women on an MPMGI diet [21]. Collectively, these observations suggest that while an HPLGI diet may have a positive impact on maternal outcomes, such as limited GWG and reduced pregnancy complications [19], it may have an unfavorable impact on long-term offspring outcomes. However, differences in BMI z-scores between the two APPROACH groups did not reach statistical significance [21], consistent with our findings on BMI z-scores in early childhood from the present study.

It is well documented that elevated maternal blood glucose levels are associated with excessive fetal growth, cord blood serum C-peptide, as well as other adverse pregnancy outcomes [34, 35]. Implementing a low GI diet during pregnancy has been associated with reduced birth weight, decreased incidence of macrosomia, and lower risk of offspring being born large-for-gestational-age among women with a high risk of gestational diabetes mellitus [36]. The potential mechanisms underlying the benefits of a low-GI diet may include the attenuated rise of postprandial glucose levels, which in turn reduces hyperinsulinemia [37] and oxidative stress [38]. Studies examining the effects of low GI diets and high GI diets indicate that offspring born to women following a low-GI diet during pregnancy tend to have a lower ponderal index, a marker of adiposity at birth [39, 40]. Additionally, another study examined differences in offspring BMI z-score across GI quantiles of the maternal diet found that children born to women with higher GI intake (Q4: GI 111-1603) during pregnancy had higher BMI z-scores at 7 years compared to those in the lowest GI quantile (Q1: GI 6-63) [41]. These observations support the concept that exposure to elevated glucose levels in utero can influence fetal growth and development [42, 43]. Despite the apparent benefits of a low GI diet during pregnancy on offspring health, our study did not support these findings. One possible explanation could be the interaction between the high protein and low GI, leading to a masking effects. High-protein foods tend to have a lower GI due to their slower digestion and absorption rates. This could potentially counteract the expected benefits of low GI foods.

However, ensuring adequate maternal protein intake during pregnancy is important to ensure sufficient amino acid availability for fetal growth and development. Consequently, there is ongoing speculation regarding the impact of increased maternal protein consumption during pregnancy on fetal development. Both excessive and insufficient maternal protein intake can result in adverse pregnancy outcomes such as impaired fetal growth [44]. However, it is hypothesized that heightened exposure to protein during fetal development might impact the offspring’s future protein needs and appetite regulation, potentially leading to calorie overconsumption in an attempt to meet the ‘upregulated’ protein needs, which can lead to weight gain [45]. Additionally, the overall protein intake during infancy (from birth to 2 y) appears to be associated with later obesity outcomes during childhood or adolescence [46, 47]. This suggests that offspring with prolonged exposure to high protein during infancy, and potentially exposure to high protein in their early environment, may be associated with the development of overweight and obesity in the long term. The association between an HPLGI diet during pregnancy with higher offspring body weight and BMI at 18 years, but not earlier in life, is noteworthy. This pattern may reflect a latent effect of maternal nutrition that only emerges during late adolescence, a period marked by significant hormonal and metabolic changes, presenting a challenge to metabolic homeostasis [48]. Similarly, this delayed effect has been observed with other early-life exposures, such as breastfeeding [49]. In another Danish cohort, an association between protein intake (substituted for carbohydrate) during pregnancy and the risk of offspring being overweight at 19–21 years of age was found [18], consistent with our findings at 18 years in the DNBC. Thus, the possibility exists that the influence of protein exposure in utero on the risk of obesity in the offspring manifests later in life, potentially explaining the higher weight and BMI observed in our study at 18 years. On the other hand, adequate protein intake is needed for the regulation of muscle mass, which is a major component of fat-free mass. In the Generation R study, including 2694 Dutch mother-offspring dyads, an association was found between maternal protein intake during pregnancy and higher offspring fat-free mass index, but not BMI or fat mass index at 6 years, measured by DEXA [17]. This highlights the limitation of BMI and BMI z-score as these metrics do not account for body composition, particularly when considering the impact of high protein diets on fat-free mass [50]. Moreover, while our primary focus was on protein intake and glycemic index, other dietary components may have contributed to the observed associations. For instance, lower fiber intake—often associated with high-protein, low-glycemic-index diets—may influence long-term metabolic outcomes through mechanisms involving gut microbiota, insulin sensitivity, and appetite regulation [51]. These mechanisms could contribute to an unfavorable intrauterine environment, potentially predisposing offspring to increased adiposity in late adolescence.

The generalizability of the findings from the DNBC may be limited due to lifestyle differences in the late 1990s, notably lower rates of obesity, and lower intakes of protein. In the present study, women in the HPLGI group had higher pre-pregnancy BMI and significantly lower energy intake compared with women in the MLMGI group, while gaining the same weight during pregnancy. It is well-documented that higher BMI is associated with a higher tendency for underreporting [52]. If indeed the HPLGI group is underreporting their dietary intake, this could potentially introduce a bias regarding the percent contribution of protein to total energy. Furthermore, all dietary data were self-reported in an FFQ by the women in GW 25. To enhance accuracy, the most extreme measures of energy intake were excluded, thus reducing the magnitude of measurement error. Moreover, offspring height and weight measurements were self-reported at 7 years, 11 years, 14 years, and 18 years by questionnaires, but the extent of potential misreporting in weight may have been less apparent due to the varying time intervals between the initial exposure (during pregnancy) and subsequent assessments (from birth to 18 years of age). However, this does not negate the possibility that such factors could be linked to misreporting. Additionally, environmental and lifestyle factors throughout childhood and adolescence may have influenced growth trajectories, potentially attenuating, amplifying, or delaying the associations with maternal diet. However, detailed data on these factors were not available for inclusion in the present analysis. We acknowledge that GWG and gestational age may lie on the causal pathway, and adjusting for them can introduce bias. However, the inclusion of these variables in the fully adjusted model (Model 3) was intended to isolate the association between the HPLGI diet and offspring outcomes, minimizing the influence of these potentially confounding factors. Despite these limitations, this cohort is a unique source of valuable longitudinal data with repeated measurements that allowed us to use detailed dietary information collected during pregnancy and offspring markers of adiposity from birth to the age of 18 years, while controlling for potential confounders.

In summary, this population-based cohort study suggests that offspring born to women who consumed an HPLGI diet during pregnancy exhibited higher body weight and BMI at 18 years of age compared to those born to women consuming an MPMGI diet. Further research may be needed to explore these findings and clarify the potential mechanisms and implications of maternal diet during pregnancy on offspring health outcomes.

## Supplementary information

Supplementary material

## Data Availability

Data sharing is restricted to uphold the privacy protection of participants within the Danish National Birth Cohort. Data are available from The Danish National Birth Cohort Secretariat, e-mail: bsmb@ssi. dk, for researchers who meet the criteria for access to confidential data.
