# Supplementary material for: Association of a high-protein and low-glycemic-index diet during pregnancy with offspring growth and obesity until the age of 18 years – a target trial emulation
Source: Eur J Clin Nutr. 2025 Sep 25;79(11):1121–9. doi: 10.1038/s41430-025-01666-2 (PMC12580314; doi:10.1038/s41430-025-01666-2)
Supplement: Supplementary file 1 — Supplementary material [file 41430_2025_1666_MOESM1_ESM.docx]

**Association of a high-protein and low-glycemic-index diet during pregnancy with offspring growth and obesity until the age of 18 years – a target trial emulation**

**Christina Sonne Mogensen^1^**, Christian Mølgaard^1^, Faidon Magkos^1^, Nina Rica Wium Geiker^2^, Anne Ahrendt Bjerregaard^3,5^, Charlotta Granström^3^, Thorhallur Ingi Halldorsson^3,4^, Sjurdur Frodi Olsen^3,6,7,8^.

^1^ Department of Nutrition, Exercise and Sports, Faculty of Science, University of Copenhagen (UCPH), Copenhagen, Denmark

^2^ Centre for Childhood Health, Copenhagen, Denmark

^3^ Department of Epidemiology Research, Statens Serum Institut, Copenhagen, Denmark

^4^ Faculty of Food Science and Nutrition, School of Health Sciences, University of Iceland, Reykjavik, Iceland

^5^ Section of Epidemiology, Frederiksberg Hospital Center for Klinisk Forskning og Forebyggelse, Frederiksberg, Denmark

^6^ Department of Public Health, University of Copenhagen, 1353 Copenhagen, Denmark

^7^ Harvard TH Chan School of Public Health, Boston, MA

^8^ University of the Faroe Islands, J. C. Svabos gøta 14, Torshavn, The Faroe Islands

**Corresponding author:** Christina Sonne Mogensen, Department of Nutrition, Exercise and Sports, Faculty of Science, University of Copenhagen, Rolighedsvej 26, DK-1958 Frederiksberg C, Denmark, Phone: +45 3533 3284, Email: [csm@nexs.ku.dk](mailto:csm@nexs.ku.dk)

**Table S1: Maternal and offspring characteristics of those who were retained in the study from birth to 18 years of age (Complete) and those who lost to follow-up before the 18-year follow-up (Dropouts)**

|  | N | Complete  (N=3402) |  | N | Dropouts  (N=3577) | P-value |
| --- | --- | --- | --- | --- | --- | --- |
| Maternal age (years) | 3402 | 30.6 (4.1) |  | 3577 | 30.3 (4.2) | **0.001** |
| Maternal weight (kg) | 3402 | 81.5 (11.5) |  | 3577 | 81.9 (11.7) | 0.175 |
| Maternal height (cm) | 3402 | 168 (6.0) |  | 3577 | 168 (6.1) | 0.817 |
| Maternal pre-pregnancy BMI  (kg/m^2^) | 3402 | 28.7 (3.5) |  | 3577 | 28.8 (3.6) | 0.071 |
| GWG (kg) | 2810 | 13.5 (7.0) |  | 2884 | 13.5 (7.1) | 0.794 |
| Maternal energy intake (kJ) | 3402 | 9831 (2211) |  | 3577 | 9705 (2259) | **0.018** |
| Maternal Protein intake (%) | 3402 | 15.1 (1.95) |  | 3577 | 15.2 (2.02) | 0.647 |
| Maternal Glycemic index  (pr. day) | 3402 | 74.4 (14.8) |  | 3577 | 74.0 (15.0) | 0.255 |
| Gestational age (days) | 3402 | 281 (12) |  | 3577 | 280 (13) | **0.016** |
| Offspring weight (kg)^$^ | 3402 | 3.69 (0.56) |  | 3577 | 3.68 (0.60) | 0.659 |
| Offspring BMI z-score^$^ | 3385 | -0.12 (1.14) |  | 3545 | -0.17 (1.18) | 0.051 |

Values are presented as mean (SD). Bold indicates a significant difference between the groups. ^$^Outcomes at birth.
